# Desmoplastic infantile ganglioglioma: Report of a case and review of the literature

**DOI:** 10.4103/1817-1745.66669

**Published:** 2010

**Authors:** Bita Geramizadeh, Ahmad Kamgarpour, Ali Moradi

**Affiliations:** Department of Pathology and Transplant Research Center, Shiraz University of Medical Sciences, Iran; 1 Department of Pathology Surgery, Shiraz University of Medical Sciences, Shiraz, Iran

**Keywords:** Desmoplastic infantile ganglioglioma, histopathology, immunohistochemistry

## Abstract

Desmoplastic infantile ganglioglioma (DIG) is a rare supratentorial brain tumor occurring mostly before the age of 2 years. It has a good prognosis and total excision of the tumor is curative, necessitating no further treatment. An accurate pathologic diagnosis is crucial. Until now, <60 cases of this tumor type have been reported. Herein, we report a 3-month-old boy with intractable seizure who was found to have DIG after surgery.

## Introduction

Desmoplastic infantile ganglioglioma (DIG) was first described by Vanderberg in 1987.[1] Before his introduction, similar tumors were labeled as composite cerebral neuroblastoma and astrocytoma.[2]

Now, this tumor is recognized as a distinct entity and is included in the World Health Organization (WHO) classification under the category of neural and mixed glio-neuronal tumors, and is in the grade I of the WHO classification.[3] Until now, <60 cases of DIG have been described.[4]

We present a case in a 3-month-old male infant presenting with intractable seizure and a large mass in the right temporal lobe, which showed classic histological features of DIG. Hence, the current case extends the reported spectrum of this rare tumor and helps pathologists in considering the diagnosis especially in young patients.

## Case Report

**Figure 1:**
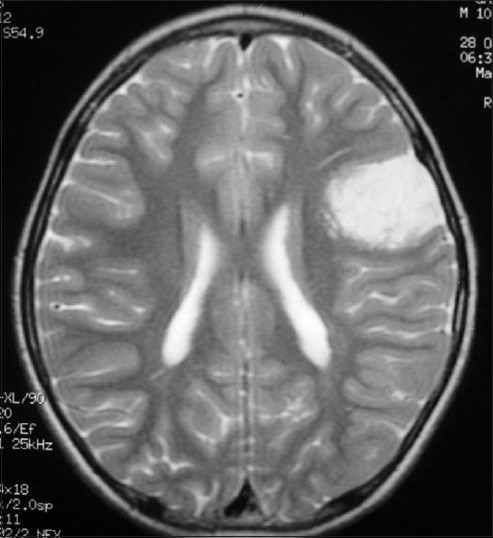
Brain MRI scan shows a large superficial and temporal mass

A 3-month-old infant was admitted with the chief complaint of seizure. He was the first child of the family, born during an uncomplicated full-term pregnancy and a normal vaginal delivery. The family history was unremarkable. Physical examination revealed no abnormality, with a normal neurological examination. Magnetic resonance imaging (MRI) scan showed a hypodense area in the right temporal region, with marked enhancement in the medial parts and severe surrounding brain edema [Figure 1].

A right temporal craniotomy accompanied by peripheral temporal lobectomy was performed and more than 90% of the tumor was excised. The postoperative period was uneventful, with no seizure after surgery.

**Figure 2:**
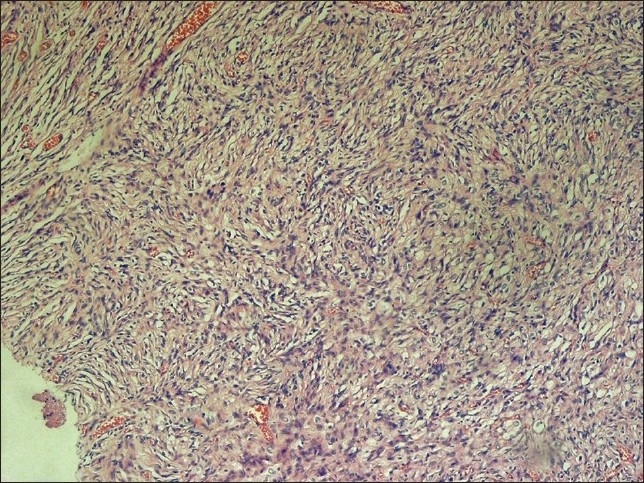
Low power view of the sections from brain tumor shows severe desmoplasia and fascicular pattern of the arrangement of tumor cells (H&E, ×100)

**Figure 3:**
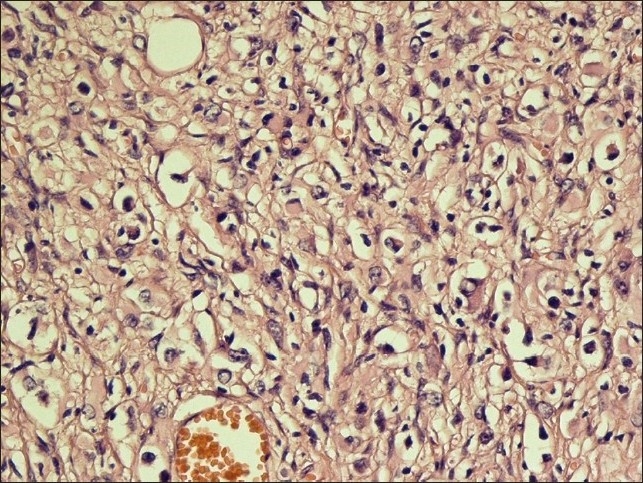
High power view shows isolated ganglion-like cells. (H&E, ×250)

The specimen, which was received in the pathology department, showed multiple fragments of grayish and firm tissue, altogether measuring about 3 cm × 3 cm × 2 cm. The hematoxylin and eosin (H and E) stain revealed a markedly desmoplastic tumor, showing deposition of dense collagen fibers. The neoplastic cell population was heterogenous, composed of spindle-shaped astrocytes with a fascicular arrangement in the abundant collagenous reticulin-rich stroma [Figure 2]. Scattered ganglion cells were also observed, indicating neuronal differentiation [Figure 3]. No mitosis or necrosis was present. A preliminary diagnosis of desmoplastic infantile ganglioglioma was made.

Immunohistochemistry (IHC) was performed to confirm the diagnosis. There was diffuse reactivity with glial fibrillary acidic protein (GFAP) in addition to focal isolated ganglion cells being positive with synaptophysin [Figure 4A and B]

**Figure 4A:**
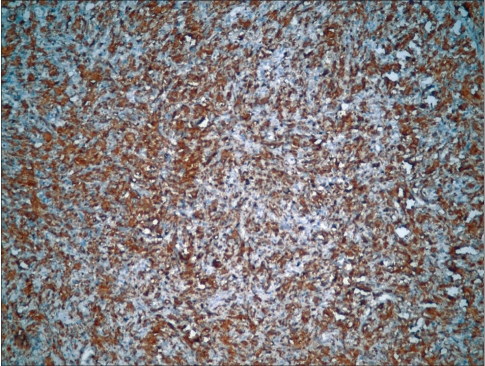
Diffusely GFAP reactive tumor cells along with focal isolated ganglion cells being positive with synaptophysin

**Figure 4B:**
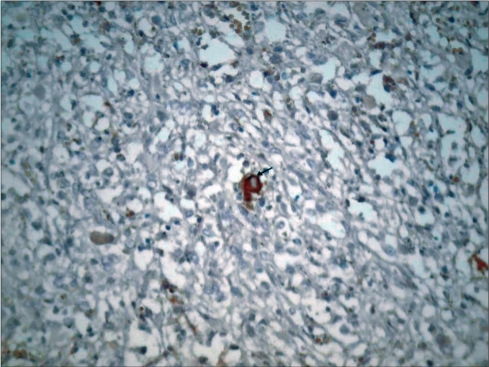
Isolated synaptophysin positive ganglion cells (arrow)

Follow-up of the patient after 6 months is unremarkable, with no seizure or any evidence of recurrence.

## Discussion

DIGs are rare intracranial tumors, most likely diagnosed in the first 24 months of life.[3]

Boys are affected more commonly than girls.[4] Symptoms of DIG are intracranial hypertension, sunset eye, enlarging head circumference, bulging fontanels, variable localizing signs, including seizures, or paresis.[5]

They are massive, firmly attached to the dura, extensively infiltrate the subarachnoid space but do not involve the ventricular system.[6] Most commonly, CT scan and magnetic resonance imaging show a large superficial large cerebral mass with solid and cystic areas.[4] The solid component of the tumor frequently shows contrast enhancement. Calcification has not been reported in imaging studies.[3]

Pathologic features of the tumor have been clearly described in several previous reports. Histologically, the most prominent feature of DIG is desmoplasia and spindle cells with a storiform pattern of arrangement.[5] There is also a ganglion cell component, which is present as single cells or clusters.[7]

The first component can be shown to be GFAP positive, but the latter component is of neuroepithelial origin and reactive with markers such as synaptophysin.[7]

The main histologic differential diagnoses are reticulin-rich desmoplastic tumors such as pleomorphic xanthoastrocytoma, which can be differentiated by age of the patient, prominent lipidization of the cells and absence of neural component.[6] Another tumor in this category is gliofibroma, which is infratentorial and lacks the neural component.[^9^] Age of the patient and lack of desmoplasia in the ganglioglioma can differentiate this tumor from DIG.[6]

Our case was a 3-month-old baby with intractable seizure of undetermined cause. CT showed a large superficial tumor in the temporal lobe, which is the third most common site of brain involvement. Pathology of the tumor revealed typical histologic and IHC features.

Complete resection is usually curative with no further additional therapy.[8] Our case is also well after tumor resection and now, after 6 months, has no evidence of tumor progression or recurrence.
